# Solving Math Problems Approximately: A Developmental Perspective

**DOI:** 10.1371/journal.pone.0155515

**Published:** 2016-05-12

**Authors:** Dana Ganor-Stern

**Affiliations:** Department of Psychology, Achva Academic College, Achva, Israel; University of Leuven, BELGIUM

## Abstract

Although solving arithmetic problems approximately is an important skill in everyday life, little is known about the development of this skill. Past research has shown that when children are asked to solve multi-digit multiplication problems approximately, they provide estimates that are often very far from the exact answer. This is unfortunate as computation estimation is needed in many circumstances in daily life. The present study examined 4^th^ graders, 6^th^ graders and adults’ ability to estimate the results of arithmetic problems relative to a reference number. A developmental pattern was observed in accuracy, speed and strategy use. With age there was a general increase in speed, and an increase in accuracy mainly for trials in which the reference number was close to the exact answer. The children tended to use the sense of magnitude strategy, which does not involve any calculation but relies mainly on an intuitive coarse sense of magnitude, while the adults used the approximated calculation strategy which involves rounding and multiplication procedures, and relies to a greater extent on calculation skills and working memory resources. Importantly, the children were less accurate than the adults, but were well above chance level. In all age groups performance was enhanced when the reference number was smaller (vs. larger) than the exact answer and when it was far (vs. close) from it, suggesting the involvement of an approximate number system. The results suggest the existence of an intuitive sense of magnitude for the results of arithmetic problems that might help children and even adults with difficulties in math. The present findings are discussed in the context of past research reporting poor estimation skills among children, and the conditions that might allow using children estimation skills in an effective manner.

## Introduction

Past research on numerical cognition has focused on how people solve arithmetic problems exactly and how this ability develops with age (e.g., [1–3]). Relatively little work was devoted to computational estimation, which is the process of producing an approximate answer to an arithmetic problem, and to its development. The dearth of research on this topic is unfortunate because of the following reasons. First, computation estimation is an important skill in everyday life. It is needed when a person is faced with an arithmetic problem but exact calculation is not possible due to time or attentional limitations. In such a case, computational estimation might be used instead. It is also useful as a "sanity check", to quickly evaluate whether the result obtained through the use of a calculator or mental calculation is reasonable. Second, past research has shown that children are poor estimators, and even many adults are not good at it (see [4] for a review). Thus, exploring this topic will extend our understanding of the numerical processes taking place in everyday life, and perhaps propose ways to improve such important skills.

Most past research on computational estimation has used the *estimation production task*, in which participants are presented with an arithmetic problem and are asked to estimate how big the answer to the problem is without calculating it exactly (e.g., [5, 6, 7]). The estimation production task requires both conceptual and procedural knowledge. The conceptual knowledge includes the simplification principle—the understanding that simplifying a problem to an approximate solution is legitimate in some circumstances, and the proximity principle—the understanding that an estimate should be as close as possible to the exact answer [5]. The procedural knowledge relevant exclusively for estimation includes the ways to simplify a problem in order to solve it approximately and the compensation procedures to correct for the distortion produced by the simplification procedures. Children acquire the simplification principle and the relevant rounding procedures around 4^th^ or 5^th^ grade, and the proximity principle along its procedures even later, around the age of 15 [5, 8].

The evaluation of estimation skills is usually based on accuracy measures. Estimation is expected to produce faster responses than exact calculation but at the expense of accuracy. The question of how accurate an estimate should be to be considered accurate enough is a practical question, and the answer probably depends on the context. It seems to be agreed by all however, that estimates that are off by an order of magnitude (i.e., place value errors) are unacceptable. Such errors are especially common in estimates of results of multiplication problems. For example, providing an estimate of 320 for the problem 85 x 43 is considered a place value error. When using a set of 2 digit (D) x 2D multiplication problems, Ganor-Stern and Siegler [9], reported 52%, 28%, and 9% place value errors for 6^th^ graders, 8^th^ graders and adults, respectively. Such errors might reflect weak procedural or conceptual knowledge, or a working memory limitation. Importantly, in the majority of the cases, the estimates involving place value errors were underestimates, that is they were ten times smaller than the exact answer.

In addition to looking at accuracy of estimates, past research has also examined the strategies used when producing estimates to arithmetic problems. The common strategies involve rounding procedures. For example, in the context of 2-digit numbers multiplication, the common rounding procedures were rounding either one or both multiplicands *down* to the nearest decade, or rounding one multiplicand up and the other one down to the nearest decade. In some cases participants used post-compensation to correct for the error introduced by the rounding procedure. That is, if they rounded both multiplicands down, they would add some to the product to correct for the error introduced by the rounding down. Importantly, such post-compensation procedures were used often by adults but were rarely used by children [5–7, 10].

Thus, the emerging picture is that the estimation production task demonstrates the children’s procedural knowledge on the rounding procedures, but the prevalence of their place value errors might suggest weak sense of magnitude or limited working memory resources. The children’s performance in this task could reflect poor general estimation skills. If so, it could raise doubt whether estimation can be used effectively as a substitute for exact calculation, or as a sanity check for the solutions obtained through other means. Alternatively, it might reflect the inability to *produce* estimates rather than poor estimation skills in general.

To address this issue performance on a different computational estimation task should be explored. In the *estimation comparison task* a multi-digit multiplication problem is presented together with a reference number and participants are asked to indicate whether they estimate the answer to that problem to be larger or smaller than the reference number [11, 12].

Performance in terms of speed and accuracy was found to decrease for larger multiplicands (i.e., the problem size effect). It was enhanced for reference numbers that were far vs. close from the exact answer (i.e., the ratio or distance effect). It was also enhanced for smaller vs. larger reference numbers (i.e., the reference number size effect). For example, it was easier for the participants to decide that the exact answer for a problem such as 37 x 54 was larger than 400 than it was smaller than 4000 and to decide that it was smaller than 8000 than it was smaller than 4000 [12, 13]. The ratio/distance and size effects in quantity and magnitude comparisons suggest the existence of a condensed approximate representation. These effects are considered the markers of the ANS (Approximate Number system) (e.g., [14]). The ANS is a mental system of approximate magnitude representation that is universal, and appears early in development. This system is assumed to be active whenever a numerical process is taking place involving either symbolic or nonsymbolic stimuli (e.g., [14]). The presence of the ratio/distance and size effects in this task might be interpreted as reflecting the involvement of the ANS in this task [12, 13].

A study using a version of this task, in which young children were presented with non-symbolic stimuli found a similar pattern. McCrink and Spelke [15] examined the ability of 5- to 7-year-old children to estimate the result of multiplication of numerosities relative to a reference quantity. Their performance was above chance level, and it was affected by the ratio between the correct and proposed answer. The fact that the children were prior to any formal schooling suggests that their performance relies on a core, intuitive multiplication ability which is based on their ANS.

As to the underlying processes, Ganor-Stern [12, 13] identified two main strategies used by college students to solve this task. The first is an approximate calculation strategy—rounding either one or two multiplicands, multiplying the rounded numbers, and comparing the product to the reference number. An example of a self-report of this strategy for the problem 54 x 36 and the reference number of 400, might be: "It is like 50 x 40, so it is larger than the reference number". This method produces relatively high accuracy but at the cost of slower responses. It is assumed to require rounding and multiplication skills as well as working memory resources [16]. The second strategy is the sense of magnitude strategy—basing the response on an intuitive sense of magnitude without any calculation. Thus, for the same problem, if the reference number is 100, participants might indicate that "it feels much larger than this number". This strategy reflects an intuitive coarse sense of magnitude, which is presumably built on the individual life experience with multiplication. Practice in solving arithmetic problems leads to sensitivity to various aspects of these problems, (e.g.,[17, 18, 19]), one of which is the range of magnitudes possible for a specific type of problems. This intuitive sense of magnitude is further fine-tuned by the experience with the specific set of problems presented during the experiment [20]. This intuitive coarse sense of magnitude might be linked to the ANS. This strategy, in contrast to the former, is associated with less accurate responses, especially when the reference number is close, but it is faster and requires little working memory resources. A recent study using eye movement measures validated the existence of these two distinct strategies, by showing different eve movements patterns for the two strategies [20]. When the participants used the approximate calculation strategy they focused longer on the multiplicands than on the reference number, while when they used the sense of magnitude strategy their looking time was distributed equally between the multiplicands and the reference number.

The approximate calculation strategy was used more often by adults than the sense of magnitude strategy. However, most participants used both strategies, consistent with past studies (e.g., [21]). Importantly, the participants exhibited adaptivity in strategy choice [21–23], as they used the time-consuming approximate calculation strategy more often when the reference number was close vs. far from the exact answer, and thus the faster sense of magnitude strategy was less likely to produce a correct response [12, 13].

The results were interpreted as reflecting a two-stage problem solving process, in the first stage participants attempt to reach an answer using their intuitive sense of magnitude for the results of such multiplication problems. The second is a more time-consuming approximate calculation process, which took place mainly when the first stage could not provide a definitive response, such as when the reference number is close to the exact answer [12, 13].

## Present Study

**Fig 1:**
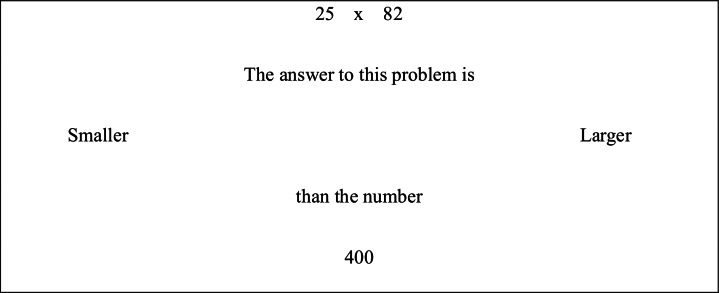
Format of an estimation comparison trial.

The goal of the present study was to examine whether the poor estimation skills exhibited by children in the estimation production task reflect poor general estimation skills, perhaps due to imprecise numerical representations, or a more task-specific weakness. For this purpose the estimation comparison task was used. To investigate development across age, 4^th^ graders, 6^th^ graders, and college students completed this task with a set of 2D x 2D multiplication problems. In each trial a multiplication problem was presented horizontally on the computer screen with a reference number below it (Fig 1). Participants had to estimate whether the answer to the problem was larger or smaller than the reference number, and to press the corresponding key. As in Ganor-Stern [12], the reference numbers were either larger or smaller than the exact answer, and they were either far (i.e. fifth or 5 times the exact answer) or close (half or twice the exact answer) to the exact answer. To explore strategy use, participants also provided self-reports and described how they reached their answer.

The following predictions regarding accuracy, speed and strategy use were tested.

### Accuracy and speed

Performance in terms of speed and accuracy should improve with age [4, 5]. Moreover, as mentioned earlier Ganor-Stern and Siegler [9] reported that in the estimation production task, about half of the estimates produced by 6^th^ graders were ten times smaller than the exact answer. If this poor performance reflects poor estimation skills in general, one might expect that the children’s accuracy in the estimation comparison task would also be very low, even approaching chance level.

### Strategy use

With age there should be more variability in strategy use and more adaptivity in strategy choice [21, 23]. Furthermore, with respect to the frequency of use of the two main strategies found in this task–the approximate calculation and the sense of magnitude, the following two possibilities were raised. Due to the training in multiplication and in rounding procedures given in school and the emphasis given to solving mathematical problems using calculation, children might be more inclined to use the approximate calculation strategy than adults, who in turn might rely more on the sense of magnitude strategy at least for far reference numbers. Alternatively, due to the children’s relatively weak multiplication skills and limited working memory resources, they might rely more often on the sense of magnitude strategy than adults, who due to their life long experience with arithmetic and enhanced working memory resources, might use mainly the approximate calculation strategy.

## Method

### Participants

Ninety-two participants took part in this study, thirty-two 4^th^ graders, thirty 6^th^ graders, and thirty college students. Four 4^th^ graders, two 6^th^ graders and two college students were excluded from the analysis due to chance level performance or an exceptionally long response time, leaving 28 participants in each age group. The mean age was 24.0 years for the adults group (17 females), 11.1 years for the 6^th^ grade group (18 females), and 9.35 years for the 4^th^ grade group (14 females). The children were from two public elementary schools in the south of Israel. The college students were from Achva Academic College and Ben-Gurion University of the Negev and they participated in the experiment for payment ($5 each). Two research assistants administered the experiment in a quiet room in school or in the laboratory.

### Ethics statement

The procedure was approved by the ethics committees of the Israeli Ministry of Education and of Achva Academic College. The college students provided written informed consent to participate in this study. Adhering to the policy of the Ministry of Education IRB, the parents of the school children denied consent by returning an enclosed form.

### Apparatus

The experiment was conducted on a Lenovo Thinkpad laptop computer with a 17-inch screen. The software was programmed in E-Prime [24].

### Stimuli

The stimuli set was composed of forty 2D x 2D multiplication problems. Following Ganor-Stern [12], the problem set was constructed with the following restrictions. There were no tie problems. No operand had 0 as units digit. No reversed orders of operands were used (53 x 76 was not used with 76 x 53). The operands ranged between 13 and 95, and the exact answers were in the range of 768–8178. In half of the problems the larger operand was on the right, while in the other half the larger operand was on the left. Each problem was associated with 4 reference numbers: one which was about one fifth of the exact answer, one which was about one half of the exact answer, one which was about twice the exact answer, and one which was about 5 times the exact answer. Reference numbers were rounded to the nearest hundred. The problems were arranged in four lists that were counterbalanced across participants. Thus, each participant responded to only one list. Within each list, each problem appeared once; across lists, each problem appeared with each of the four reference numbers. Within each list, in half of the trials the exact answer was larger than the reference number, and in the other half it was smaller than the reference number.

### Procedure

The experiment was conducted individually. The participant sat about 50 cm from the computer screen. In each trial a multiplication problem appeared on the screen with a reference number below it (see Fig 1). Participants were asked to estimate whether the answer for each problem was larger or smaller than the reference number. They had to press the "L" key if they estimated it to be larger than the reference number, and the "A" key if they estimated it to be smaller. The participants were given two examples of the task, together with the corresponding correct responses, to make sure that they understood the task requirements. Participants were explicitly told that they should not solve the problems exactly, but should only estimate whether the answer was larger or smaller than the given number. The numbers remained on the screen until the participants’ response. The order of trials was random. Participants were not allowed to use calculators or paper and pencils for calculation. Participants did not receive any feedback for their responses. In the first 8 trials, participants responded by pressing the computer keys only, while in the remaining 32 trials, after they pressed the response key for each trial, they were asked to describe how they reached their decision. The experimenter documented their descriptions.

## Results

The results section includes analyses of accuracy, speed, and strategy use.

### Accuracy and speed analysis

Analyses of variance (ANOVA’s) with age (4^th^ graders, 6^th^ graders, and adults) as a between-participants variable and distance between the exact answer and the reference number (far, close), and size of the exact answer relative to the reference number (exact answer > reference number, exact answer < reference number) as within-participants variables were conducted on percent error (PE) and response time (RT) of correct responses. Partial eta squared values (*η*^*2*^_*p*_) were calculated as SSeffect / (SSeffect + SSerror). Responses that took longer than 2.5 standard deviations above each participant mean were excluded from the analyses (less than 3% of the trials).

#### Accuracy

**Fig 2:**
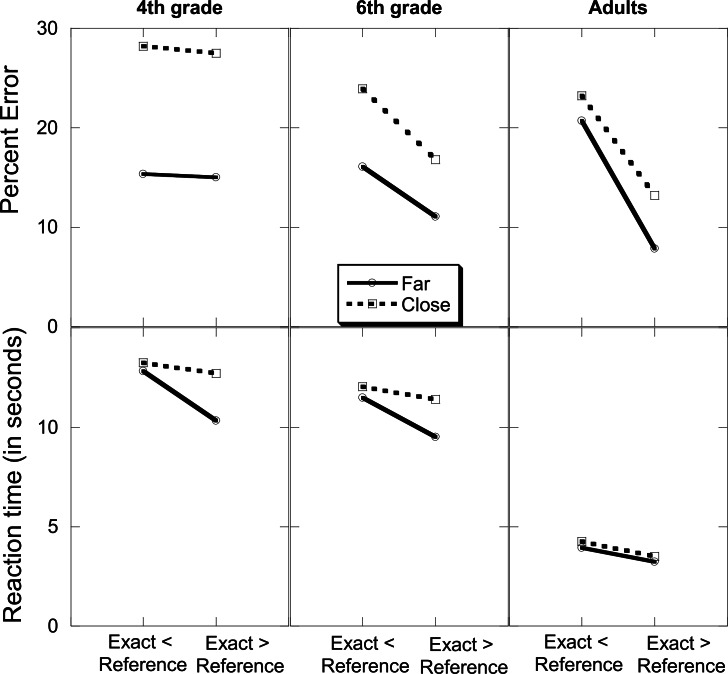
Percentage of errors (top panel) and reaction time (bottom panel) by age, the relation between the magnitude of the exact answer and the reference number and their relative distance.

As can be seen in Fig 2 (top panel), PE was lower when the exact answer was far from the reference number (14.34%) compared to when it was close to it (22.14%), *F*_(1, 81)_ = 32.44, *MSE* = 157.4, *p* = .0001, *η*^*2*^_*p*_ = .29. PE was also lower when the exact answer was larger than the reference number (15.24%) compared to when it was smaller than it (21.25%), *F*_(1, 81)_ = 5.78, *MSE* = 525.7, *p* = .02, *η*^*2*^_*p*_ = .07. PE decreased with age, *F*_(2, 81)_ = 2.38, *MSE* = 384.1, *p* = .07, *η*^*2*^_*p*_ = .06 although the effect was only marginally significant. PE was 21.52%, 16.96%, and 16.25% for the 4^th^ graders, 6^th^ graders, and adults, respectively. The age and distance variables interacted, *F*_(2, 81)_ = 3.54, *MSE* = 157.4, *p* = .03, *η*^*2*^_*p*_ = .08, as PE in the close condition decreased with age, *F*_(2, 81)_ = 4.31, *MSE* = 332.8, *p* = .02, *η*^*2*^_*p*_ = .10, from 27.86% for 4^th^ graders, 20.36% for 6^th^ graders, and 18.21% for the adults, while PE in the far condition did not differ across age, *F*_(2, 81)_ = 0.17, *MSE* = 208.71, *p* = .84. It was 15.17%, 13.57%, and 14.28% for 4^th^ graders, 6^th^ graders, and adults, respectively. Importantly, one-sample *t*-tests that compared accuracy to chance level (50%) found that accuracy was above chance level in all three age groups in all conditions (all *p*s < .05).

#### Speed

Similar patterns appeared in the speed analysis (Fig 2, bottom panel). RT decreased with age, *F*_(2, 81)_ = 10.51, *MSE* = 228.74, *p* = .0001, *η*^*2*^_*p*_ = .21. It was 12.28 sec for 4^th^ graders, 11.11 sec for 6^th^ graders, and 3.73 sec for the adults. Scheffe post hoc tests showed that the adults were faster than the children groups, which did not differ (p < .05). RT was shorter when the exact answer was far (8.56 sec) from the reference number compared to close (9.53 sec), *F*_(1, 81)_ = 9.78, *MSE* = 8.09, *p* = .002, *η*^*2*^_*p*_ = .87. RT was shorter when the exact answer was larger than the reference number (8.45 sec) compared to when it was smaller than it (9.63 sec), *F*_(1, 81)_ = 14.11, *MSE* = 8.32, *p* = .001, *η*^*2*^_*p*_ = .15. The two variables interacted, *F*_(1, 81)_ = 4.69, *MSE* = 5.26, *p* = .03, *η*^*2*^_*p*_ = .57, indicating that the difference in RT between close and far trials was larger when the exact answer was larger than the reference number. Similar to the PE analysis, the distance effect in RT was somewhat smaller for the adults compared to the children groups, although the effect did not reach significance, *F*_(2, 81)_ = 1.22, *MSE* = 8.09, *p* = .30.

### Strategy analysis

#### Classification of strategy use

The classification of participants’ self-reports into strategies was done independently by two researchers based on the experimenter verbatim, and the percentage of agreement was 97%. At the group level, the approximate calculation strategy was used in 35% of the trials among 4^th^ graders, in 47% of the trials among 6^th^ graders, and in 70% of the trials among the adults group. The sense of magnitude strategy was used in 62% of the trials among 4^th^ graders, in 50% of the trials among 6^th^ graders, and in 25% of the trials among the adults group. The rest of the trials (less than 5%) could not be classified into any of those strategies and thus were not analyzed.

An individual level analysis classified participants as consistent users of a certain strategy if they reported using it in at least 80% of the trials [25]. The distribution of participants varied with age, χ^2^ (2) = 12.98, *p* = .01, with the main difference between the adults and the two children groups, which did not differ. As can be seen in Table 1, 21 out of the 4^th^ graders (75%) used one strategy consistently, 14 of them used the sense of magnitude strategy, and 7 used the approximate calculation strategy. Exactly the same number of 6^th^ graders employed one strategy consistently, however the distribution of strategy use differed with 10 participants implementing the approximate calculation strategy and 11 using the sense of magnitude strategy. Sixteen adults (57%) employed one strategy consistently, with 14 of them implementing the approximate calculation strategy and only two participants using the sense of magnitude strategy. Thus, both the group level information and the individual level analysis showed a clear increase in the use of the approximate calculation strategy with age.

**Table 1:** Distribution of participants by age and strategy use.

	Consistent users of the approximate calculation strategy	Consistent users of the sense of magnitude strategy	Inconsistent strategy users
4^th^ grade	7	14	7
6^th^ grade	10	11	7
Adults	14	2	12

#### Strategy choice

**Fig 3:**
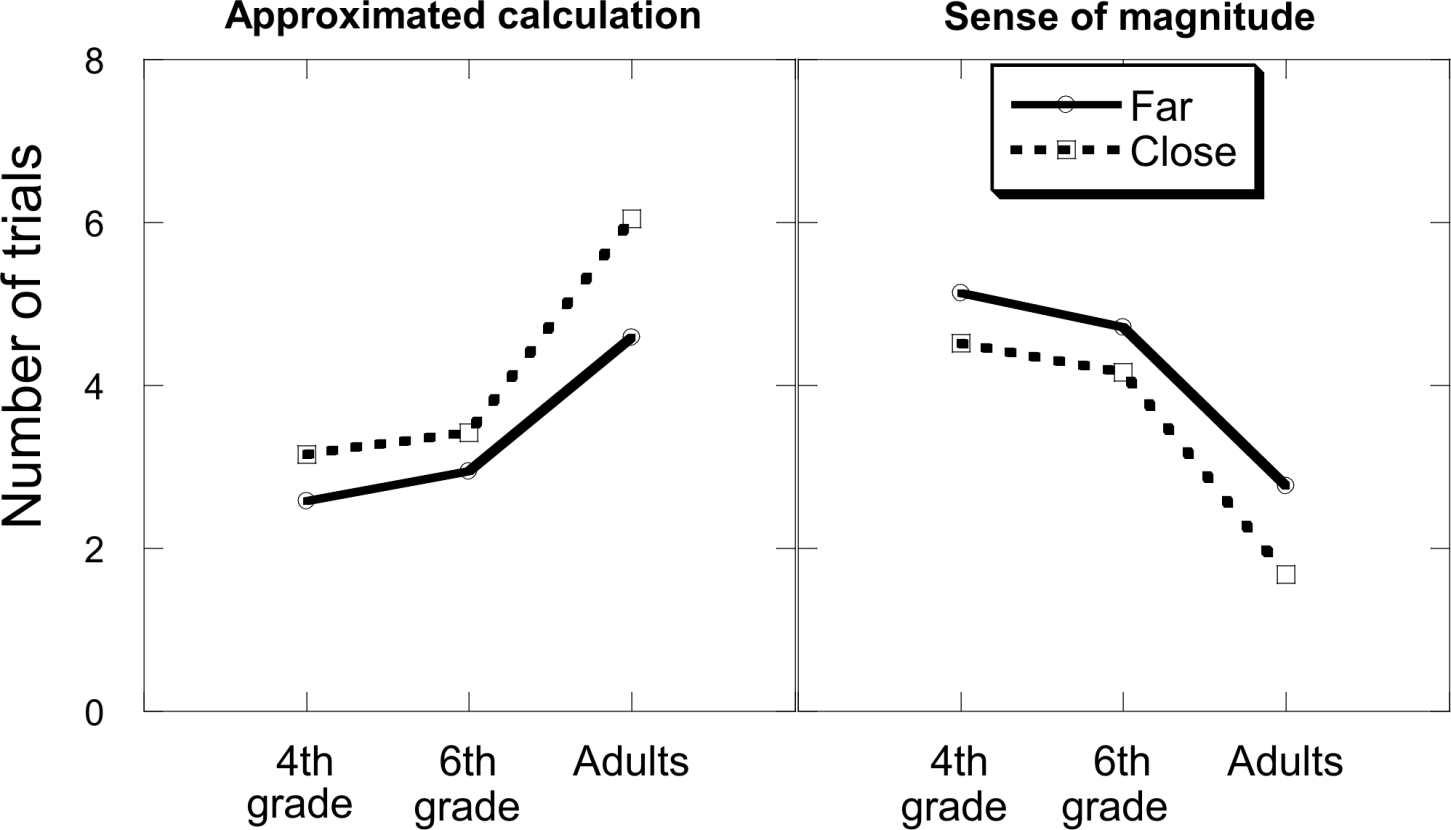
Mean number of trials that were solved by the approximate calculation strategy (left panel) and by the sense of magnitude strategy (right panel) by age and by the distance between the exact answer and the reference number.

Since most participants in all age groups used the two strategies to solve the task, the next step was to investigate how participants decided which strategy to use for which item. Thus, the frequency of use of each strategy was entered into an analysis of variance with age, strategy, size of the reference number relative to the exact answer, and distance as independent variables. This analysis was limited to the participants that used both strategies (19 4^th^ graders, 18 6^th^ graders, and 22 adults). The two effects involving the strategy variable are theoretically important (see Fig 3). There was a significant interaction between strategy and age, *F*_(2, 56)_ = 5.51, *MSE* = 54.42, *p* = .01, *η*^*2*^_*p*_ = .16, indicating that the frequency of use of the approximate calculation strategy increased with age, and that the frequency of use of the sense of magnitude strategy decreased with age. Moreover, the significant interaction between strategy and distance, *F*_(1, 56)_ = 24.24, *MSE* = 3.04, *p* = .001, *η*^*2*^_*p*_ = .30 indicated that while the approximated calculation strategy was used more often when the reference number was close (vs. far), the sense of magnitude strategy was used more often when the reference number was far (vs. close).

### Relationship between strategy use, accuracy, and speed

As mentioned in the Introduction, the two strategies are expected to produce different patterns of speed and accuracy, with the approximate calculation strategy expected to produce accurate but slow responses, and the sense of magnitude strategy expected to produce fast and less accurate responses [12, 13]. Moreover, the advantage of the approximate calculation in accuracy is supposed to be especially pronounced for close reference numbers.

To examine the effects of age and strategy use on accuracy and speed two analyses were conducted. The first looked at the accuracy and speed of participants who used a single strategy throughout the whole experiment. Among the 4^th^ graders, 3 used the approximate calculation strategy exclusively and 6 the sense of magnitude strategy solely, among the 6^th^ graders, 6 used only the approximate calculation strategy and 4 solely the sense of magnitude strategy, and among the adults, 5 used only the approximate calculation strategy and one participant used only the sense of magnitude strategy. Since there was only one adult that used the sense of magnitude exclusively the following analysis was limited to the children groups. ANOVA’s with age and strategy as between-participant variables and distance and size of the reference numbers as within-participant variables were conducted on PE and RT for correct responses.

**Fig 4:**
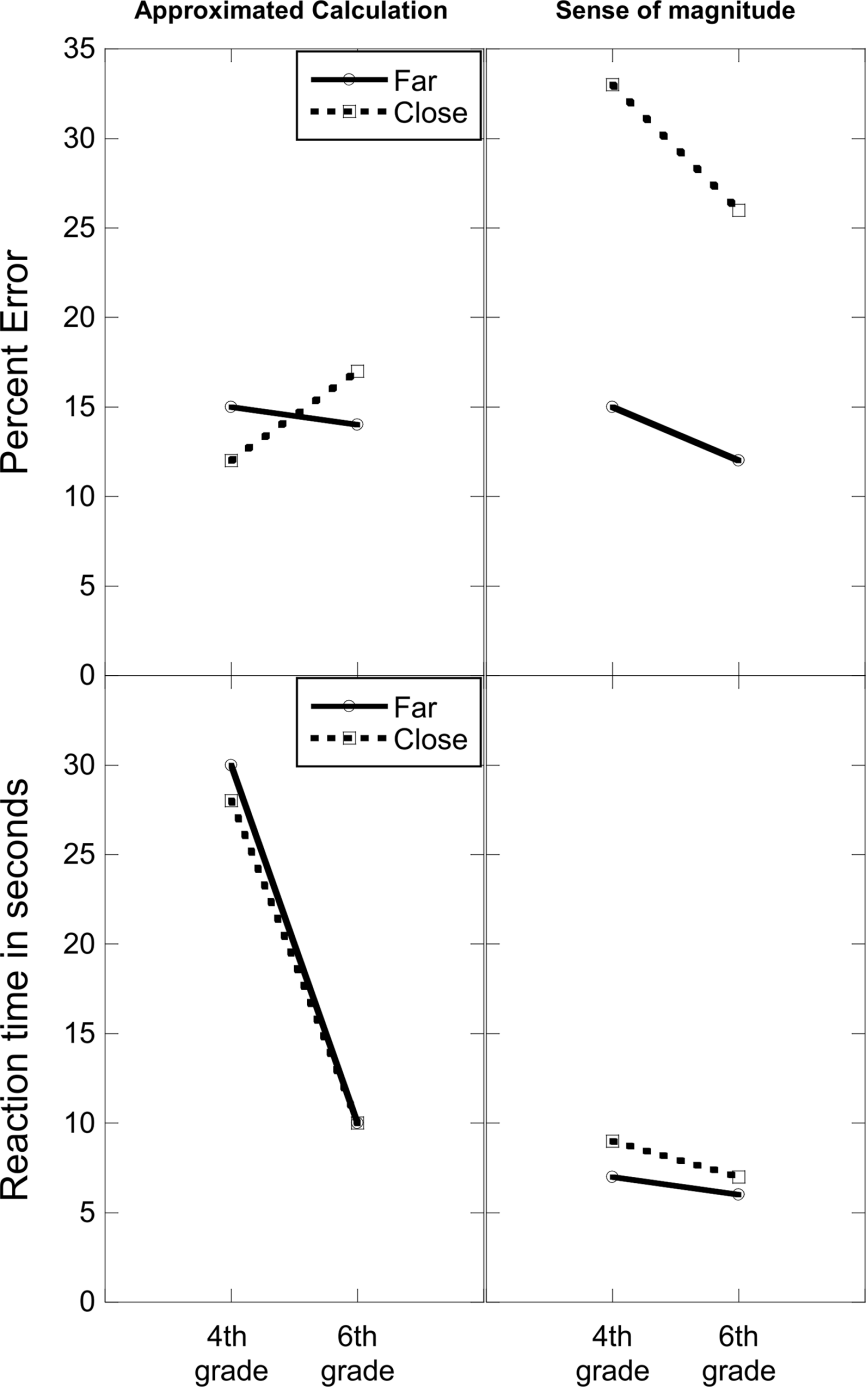
Percentage of errors (top panel) and reaction time (bottom panel) for participants who used a single strategy by strategy, age, and the distance between the exact answer and the reference number.

The PE analysis (Fig 4 top panel) has shown an interaction between strategy and distance (*F*_(1, 15)_ = 6.60, *MSE* = 170.14, *p* = .03, *η*^*2*^_*p*_ = .31, indicating that as predicted PE was higher for the sense of magnitude strategy compared to the approximate calculation strategy only when the reference number was close (*F*_(1, 15)_ = 5.81, *MSE* = 347.50, *p* = .03, *η*^*2*^_*p*_ = .28, (14.58% vs. 29.79%, for the approximate calculation and the sense of magnitude strategy, respectively), and not when it was far (14.58% vs. 13.75%), *F*_(1, 15)_ = 0.03, *p* = .85. The RT analysis (Fig 4 bottom panel) has shown that as expected, RT was shorter for the sense of magnitude strategy (6.93 sec) vs. the approximate calculation strategy (19.49 sec), *F*_(1, 15)_ = 26.56, *MSE* = 25.93, *p* = .001, *η*^*2*^_*p*_ = .63, it was also shorter for the 6^th^ (8.19 sec) vs. 4^th^ graders (18.24 sec), *F*_(1, 15)_ = 16.99, *MSE* = 25.93, *p* = .001, *η*^*2*^_*p*_ = .53. Importantly, the improvement in speed with age was larger for the approximate calculation strategy (28.95 sec for 4^th^ grade vs. 10.03 sec for 6^th^ grade) than for the sense of magnitude strategy (7.52 sec for 4^th^ grade vs. 6.35 sec for 6^th^ grade), *F*_(1, 15)_ = 13.25, *MSE* = 25.93, *p* = .001, *η*^*2*^_*p*_ = .47. The interaction between strategy and distance. *F*_(1, 15)_ = 2.93, *MSE* = 11.07, *p* = .11, *η*^*2*^_*p*_ = .16, showing that RT was faster for the far compared to the close condition only when the sense of magnitude strategy was used, although did not reach significance, is still worth mentioning as it might be informative with respect to the origin of the distance effect.

The second analysis was conducted on participants that used both strategies. It included age as a between-participant variable and strategy as a within-participant variable. Since there were not enough trials per strategy the data were collapsed across distance and size of the reference number. The speed analysis showed an age effect, *F*_(2, 46)_ = 6.09, *MSE* = 337.21, *p* < .005, *η*^*2*^_*p*_ = .21, with adults responding faster than 6^th^ graders (p < .01), and a strategy effect, *F*_(1, 46)_ = 5.53, *MSE* = 103.57, *p* < .05, *η*^*2*^_*p*_ = .11, with the responses based on the sense of magnitude strategy (9.80 sec) significantly faster than those based on the approximate calculation strategy (14.78 sec). There were no significant effects in the analysis of PE.

## Discussion

In many real life circumstances, a decision depends on a person’s ability to estimate the result of an arithmetic problem relative to a reference number. Despite the frequent use of the computational estimation skill in everyday life, relatively little is known about how people solve such tasks, and how this skill develops with age. The present research is the first to examine developmental patterns in the computational estimation comparison task by looking at the accuracy, speed, and strategy use of 4^th^ graders, 6^th^ graders, and college students.

The developmental pattern between children and adults was reflected in three measures. First, there was an increase in accuracy with age. Importantly, this increase was limited to trials when the reference number was close to the exact answer. Second, speed improved with age across conditions. Third, the dominant strategy changed with age. In 4^th^ grade the sense of magnitude was the most common strategy, in 6^th^ grade the two strategies were used equally often, while in the adults group the approximate calculation strategy was the most common. The transition from frequent use of the sense of magnitude strategy into using mainly the approximate calculation strategy with age can explain why the advantage in accuracy for the adults group was limited to the close reference numbers. The faster and less attention demanding sense of magnitude strategy is likely to produce a correct response when the reference number is far, but not when it is close.

The preference of fourth-graders to use the sense of magnitude strategy is probably due to the fact that they do not fully master the calculation skills at this stage, and thus cannot use the approximate calculation strategy effectively. Furthermore, the sense of magnitude strategy puts little burden on working memory compared to the approximate calculation strategy [16]. With age, there is an improvement in calculation skills and an increase in working memory resources [26, 27], and as a consequence more frequent use of the approximate calculation strategy. Support for this idea is provided by the sharp increase in speed found even between 4^th^ and 6^th^ graders that was especially pronounced for children who used the approximate calculation exclusively. Further rise in speed is expected between childhood and adulthood.

This increase in speed with age can explain the seemingly paradoxical pattern of adults being faster than the children although they used more often the time-consuming approximated calculation strategy. This is due to the general advantage in speed for the adults over the children, which exists across domains but is especially large when using the approximate calculation strategy.

Although children were less accurate than adults, they performed well above chance level. This finding is somewhat surprising given their performance in the estimation production task in past studies [4, 28]. As mentioned earlier, when 6^th^ graders were asked to produce estimates for a set of 2D x 2D multiplication problems, more than half of their estimates involved place value errors in contrast to less than 10% of the adults [9]. Thus, the present results provide a more optimistic picture of children’s estimation skills, and on their ability to use them as a substitute for exact calculation or as a sanity check. What could account for this difference? The presence of the reference number might be an important factor. The reference number reduces the working memory load posed by the task [16]. In addition, the reference number might serve as an anchor, which was found to improve estimates of quantities by past research [29].

Children as well as adults were affected by the characteristics of the reference number. In all age groups performance was enhanced in terms of accuracy and speed when the reference number was far vs. close to the exact answer, and when the reference number was smaller rather than larger than the exact answer [12]. These distance and size effects are considered to be the signatures of an approximate condensed representation of magnitude. Their presence in all age groups suggests the existence of a holistic representation of numbers in the thousands range even among 4^th^ graders.

The enhanced performance for trials in which the reference numbers were smaller than the exact answer might reflect the greater familiarity with the smaller numbers, which made it easier for the children as well as the adults to recognize that the exact answer should be larger than the reference number. Interestingly, the effect of familiarity on estimation is task-dependent. As mentioned earlier, when asked to produce estimates children often produced estimates that were an order of magnitude away from the exact answer. In the majority of the cases their estimates were ten times *smaller* rather than *larger* than the exact answer [9]. For example, children often estimated the answer to the problem 54 x 87 to be 400. Thus, the greater familiarity with the smaller numbers might have encouraged, especially the children, to estimate the result to be in the more familiar hundreds range. In the estimation comparison task, the greater familiarity with the smaller numbers improved participants’ discrimination when the reference numbers were smaller than the exact answer.

Children in both age groups exhibited more rigidity and less variability in strategy use compared to adults, as using a single strategy consistently was much more common among children than among adults. Such a pattern of increased variability in strategy use over development has been reported in the literature (e.g., [2, 30]). Importantly, the children as well as the adults who used multiple strategies showed adaptivity in strategy choice, as they used the approximate calculation strategy more often when the reference number was close to the exact answer and the sense of magnitude strategy more often when the reference number was far from the exact answer. This pattern was found in the past for adults who performed this task [12, 13]; the fact that it was found here even for children as young as 4^th^ graders is of importance because it suggests that children are not only sensitive to the distance between the reference number and the exact answer, they are also sensitive to the consequences of using each strategy and the need to match strategy use with problem characteristic (i.e. the distance of the reference number from the exact answer) for the sake of high accuracy. Past research has reported a similar pattern of adaptivity in strategy choice for children in computational estimation involving multi-digit addition [6, 7].

The effects of strategy use and age on accuracy and speed were examined in two ways. First when looking at children who used only a single strategy, the analysis has shown that accuracy was lower for the sense of magnitude strategy compared to the approximated calculation strategy only when the reference number was close, and that speed increased with age mainly for the approximate calculation strategy. The second analysis that focused on participants who used both strategies has shown that speed was affected by strategy and age, in an additive manner, while there were no effects on accuracy.

The difference between the results of the two analyses is possibly due to strategy choice contaminating strategy effects in the latter analysis. Participants who use multiple strategies were shown to choose which strategy to use for which item in an adaptive manner; they used the approximate calculation strategy more often for the close reference numbers, and used the sense of magnitude alone in the far condition [12, 13, 20]. This is the reason why accuracy was affected by strategy only when the strategy choice process was absent (e.g. when participants used only a single strategy for the whole experiment). Another way to dissociate effects of strategy use and strategy choice is by using the choice-no choice paradigm [21].

The present results showing that 4^th^ graders prefer the sense of magnitude strategy resemble the results of adults diagnosed with dyscalculia, who also tended to use the sense of magnitude more often than controls [13]. Together, they suggest that people with calculation problems, such as dyscalculics or those who have not yet mastered the calculation procedures well (such as 4^th^ graders), might rely on their sense of magnitude to compensate for their difficulties in calculation. This idea might be implemented in the educational setting, as suggested next.

The present study shows a developmental pattern of greater reliance on the approximate calculation strategy which reflects the application of procedures learned in school with age, and less reliance on the intuitive sense of magnitude. This pattern parallels a similar development shift that was documented in another estimation task, the number line task. In this task participants are shown a line flanked by a number at each end (e.g., 0 and 1000), and asked to place a third number (e.g., 150) on that line [31]. Numerous studies have documented a developmental shift in this task from using a logarithmic representation, which is the default basic approximate condensed magnitude representation, into using the school-taught linear representation (e.g., [17, 32, 33]). In the present study there is also evidence for a transition from using an intuitive sense of magnitude strategy into using the school-taught calculation procedures.

The present results are interpreted in the context of past works suggesting that nonsymbolic and symbolic stimuli are processed in a similar manner, possibly by the same mechanism, the ANS (e.g., [14, 34, 35]. Specifically, here the ANS was proposed to be responsible for the distance and size effects and to underlie the sense of magnitude strategy. However, alternative approaches argue that symbolic and nonsymbolic stimuli are processed separately and in a different manner. While nonsymbolic stimuli are processed approximately by the ANS, symbolic stimuli are represented exactly. This approach was recently supported by findings showing no relationship between the distance effect found for symbolic and for nonsymbolic stimuli [36].

Note that such proposals regarding the exact representation of symbolic stimuli were made with respect to numbers in the first decade [37]. The numbers used in the present context are much larger (e.g. the answers themselves are in the thousands range, while the reference numbers might be even larger) and it is very unlikely that such numbers are represented exactly. Support for the idea that the ANS underlies the distance effect and the sense of magnitude strategy is provided by the pattern showing that the distance effect in speed was present for participants that exclusively used the sense of magnitude strategy, but not for those that used the approximate calculation strategy exclusively. Although the effect did not reach significance in the present study, it was found also in past research using this task [12].

The present results together with past research using the same paradigm [12, 13, 20] suggest the existence of approximate representations of symbolic magnitudes, and an intuitive coarse sense of magnitude for the results of multiplication problems. Although the patterns seem compatible with the ANS, the symbolic approximate representation might not be based on the nonsymbolic approximate representation. Future research should explore this issue directly perhaps using brain imaging techniques.

### Implications for education

Math instruction tends to put a strong emphasis on the application of algorithmic procedures [38]. The present results together with past research on dyscalculics [13] suggest that such an estimation task that recruits the intuitive sense of magnitude might be used in addition to the algorithmic methods in order to help children with difficulties in math or with poor working memory resources. In the educational setting the teachers can give the children, prior to solving complex arithmetic problems, tasks where they judge the expected answer in comparison to a reference number. Such a prior estimation procedure might help to decrease the errors in solving complex arithmetic problems via exact calculation.

## Supporting Information

S1 Data Percent error and reaction time for correct responses by age, distance, and size of the reference number. (XLSX)
